# Integration of genomic and pharmacokinetic data to predict clinical outcomes in HIV-associated cryptococcal meningitis

**DOI:** 10.1128/mbio.01592-24

**Published:** 2024-08-27

**Authors:** Katharine E. Stott, Jason T. Mohabir, Katharine Bowers, Jennifer L. Tenor, Dena L. Toffaletti, Jennifer Unsworth, Ana Jimenez-Valverde, Ajisa Ahmadu, Melanie Moyo, Ebbie Gondwe, Wezi Chimang’anga, Madalitso Chasweka, David S. Lawrence, Joseph N. Jarvis, Tom Harrison, William Hope, David G. Lalloo, Henry C. Mwandumba, John R. Perfect, Christina A. Cuomo, The AMBITION Study Group

**Affiliations:** 1 Antimicrobial Pharmacodynamics and Therapeutics Group, Department of Pharmacology and Therapeutics, University of Liverpool, Liverpool, United Kingdom; 2 Malawi Liverpool Wellcome Clinical Research Programme, Blantyre, Malawi; 3 Broad Institute of MIT and Harvard, Cambridge, Massachusetts, USA; 4 Division of Infectious Diseases, Department of Medicine, Duke University School of Medicine, Durham, North Carolina, USA; 5 Department of Medicine, Kamuzu University of Health Sciences, Blantyre, Malawi; 6 Department of Clinical Research, Faculty of Infectious and Tropical Diseases, London School of Tropical Medicine, London, United Kingdom; 7 Botswana Harvard Health Partnership, Gaborone, Botswana; 8 Clinical Microbiology and Infectious Diseases, School of Pathology, Faculty of Health Sciences, University of the Witwatersrand, Johannesburg, South Africa; 9 Institute of Infection and Immunity, St George’s University London, London, United Kingdom; 10 Liverpool School of Tropical Medicine, Liverpool, United Kingdom; University Hospital of Cologne, Cologne, Germany

**Keywords:** *Cryptococcus*, pharmacokinetics, pharmacodynamics, cryptococcal meningitis, genomics

## Abstract

Cryptococcal meningitis causes an estimated 112,000 global deaths per annum. Genomic and phenotypic features of the infecting strain of *Cryptococcus* spp. have been associated with outcomes from cryptococcal meningitis. Additionally, population-level pharmacokinetic variability is well documented in these patient cohorts. The relative contribution of these factors to clinical outcomes is unknown. Based in Malawi, we conducted a sub-study of the phase 3 Ambition-CM trial (ISRCTN72509687), collecting plasma and cerebrospinal fluid at serial time points during the first 14 days of antifungal therapy. We explored the relative contribution of pathogen genotype, drug resistance phenotype, and pharmacokinetics on clinical outcomes including lumbar opening pressure, pharmacodynamic effect, and mortality. We report remarkable genomic homogeneity among infecting strains of *Cryptococcus* spp., within and between patients. There was no evidence of acquisition of antifungal resistance in our isolates. Genotypic features of the infecting strain were not consistently associated with adverse or favorable clinical outcomes. However, baseline fungal burden and early fungicidal activity (EFA) were associated with mortality. The strongest predictor of EFA was the level of exposure to amphotericin B. Our analysis suggests the most effective means of improving clinical outcomes from HIV-associated cryptococcal meningitis is to optimize exposure to potent antifungal therapy.

**IMPORTANCE:**

HIV-associated cryptococcal meningitis is associated with a high burden of mortality. Research into the different strain types causing this disease has yielded inconsistent findings in terms of which strains are associated with worse clinical outcomes. Our study suggests that the exposure of patients to potent anti-cryptococcal drugs has a more significant impact on clinical outcomes than the strain type of the infecting organism. Future research should focus on optimizing drug exposure, particularly in the context of novel anticryptococcal drugs coming into clinical use.

## INTRODUCTION

Cryptococcal meningitis is the most common form of meningitis among people living with HIV globally, with an estimated 152,000 cases and 112,000 deaths per annum (1). Mortality from HIV-associated cryptococcal meningitis approaches 25% at 10 weeks in resource-limited settings, even in the setting of a clinical trial and with optimized antifungal therapy (2). Clinical parameters that are predictive of mortality from HIV-associated cryptococcal meningitis include baseline fungal burden, baseline intracranial pressure, and the rate of cryptococcal clearance from cerebrospinal fluid (CSF)—termed early fungicidal activity (EFA) (3–5).

The causative pathogens of cryptococcal meningitis are members of the *Cryptococcus neoformans* and *Cryptococcus gattii* species complexes, within which several molecular types have been identified. Types VNI (encompassing VNIa, VNIb, and VNIc sublineages), VNII and VNB (encompassing VNBI and VNBII) are molecular types of *C. neoformans* var. *grubii*; VNIV corresponds to *C. neoformans* var. *neoformans*, and VNIII is the hybrid of var. *grubii* and var. *neoformans*. (6–9) In the *C. gattii* species complex, six molecular types are recognized (VGI to VGVI) (10, 11). Evidence suggests a link between molecular type and clinical outcomes, although specific associations have been inconsistent (12–16). A study of 70 clinical isolates from patients with HIV-associated cryptococcal meningitis in Botswana found that mortality outcomes were significantly worse among patients infected with VNBI strains than either VNI or VNBII strains (15). An analysis of 230 isolates collected from South African patients with HIV-associated cryptococcal meningitis revealed significantly worse survival in patients infected with VNB lineage strains compared to other strains (16). Among 140 isolates from 111 patients in Uganda, VNIII strains were associated with greater mortality than other strains (12).

In addition to inter-patient strain heterogeneity, there is evidence to suggest intra-patient strain heterogeneity. Numerous clinical studies of infection with *C. neoformans* have shown that distinct single-colony isolates from within the same episode of cryptococcosis or during relapse of infection represent different lineages, suggesting that disease can be caused by contemporaneous infection with multiple environmental strains (17–20).

Antifungal resistance is an increasing threat to the successful treatment of cryptococcal meningitis. Disomy of chromosome 1 develops in response to azole exposure and is the primary mechanism of fluconazole resistance both *in vitro* and *in vivo*, via increased copy number of the genes *AFR1* (a drug efflux pump) and *ERG11* (the fluconazole target) (21–24). For flucytosine, loss of function mutations in the genes *FCY2* (encodes cytosine permease to enable flucytosine to enter cells), *FCY1* (encodes cytosine deaminase, which converts flucytosine to its toxic metabolite 5-fluorouracil, 5FU), *FUR1* (encodes a uracil phosphoribosyltransferase responsible for downstream conversion of 5-FU to 5-FUMP to inhibit DNA and protein synthesis), and *UXS1* (encodes an enzyme that convers UDP-glucaronic acid to UDP-xylose) can all increase resistance (25, 26).

Pharmacokinetic (PK) analyses have revealed considerable variability in drug exposure within clinical populations with HIV-associated cryptococcal meningitis. This is true for all of the antifungal drugs in routine use for treatment of the condition (27–30). Detailed analyses linking the PK of antifungal drugs in patients with HIV-associated cryptococcal meningitis to pharmacodynamics (PD; as EFA) have been precluded by the use of combination antifungal therapy in modern treatment regimens so that attribution of PD effect to the PK of any one drug, in particular, is not possible. However, individual-level exposure to each drug can be estimated, and potential associations with clinical outcome parameters investigated.

In this study, we sought to investigate the relative impact of cryptococcal strain lineage, pathogen genotype, antifungal drug resistance phenotype, and antifungal drug exposure on variability in clinical outcomes in patients with HIV-associated cryptococcal meningitis. We isolated multiple cryptococcal strains from single samples of CSF, at multiple time points during clinical infection. We characterized these strains genotypically using whole-genome sequencing (WGS) and phenotypically in terms of their ability to grow *ex vivo* in the presence of the antifungal drugs fluconazole and flucytosine. We conducted a PK study and modeled those data to estimate each patient’s exposure to fluconazole, flucytosine, and amphotericin B. We then explored the relative contribution of pathogen genotype, drug resistance phenotype, and PK on PD effect and mortality.

## RESULTS

### Study population

Between November 2018 and October 2019, 64 patients were recruited to this substudy of the Ambition-CM trial from Queen Elizabeth Central Hospital in Blantyre, Malawi. In total, 31 patients were in the control arm and 33 in the single-dose liposomal amphotericin arm. The median age was 36 years (interquartile range 33–41 years) and 37% of patients were female. Median weight was 50 kg (interquartile range [IQR] 47–56 kg) and median creatinine clearance was 105.15 mL/min at enrolment (IQR 80.63–123.05 mL/min). In this highly immunosuppressed cohort, the median CD4 cell count was 39 cells/mm^3^ (IQR 21–83 cells/mm^3^, *n* = 57).

Blood samples for PK were collected on days 1 and 7 of the study at 0, 2, 4, 7, 12, and 23 h after drug administration. CSF was sampled via lumbar puncture on days 1, 7, and 14 and also opportunistically if additional lumbar punctures were required to manage raised intracranial pressure. In total, the data set included 577 plasma samples and 207 CSF samples for PK analysis.

**Fig 1:**
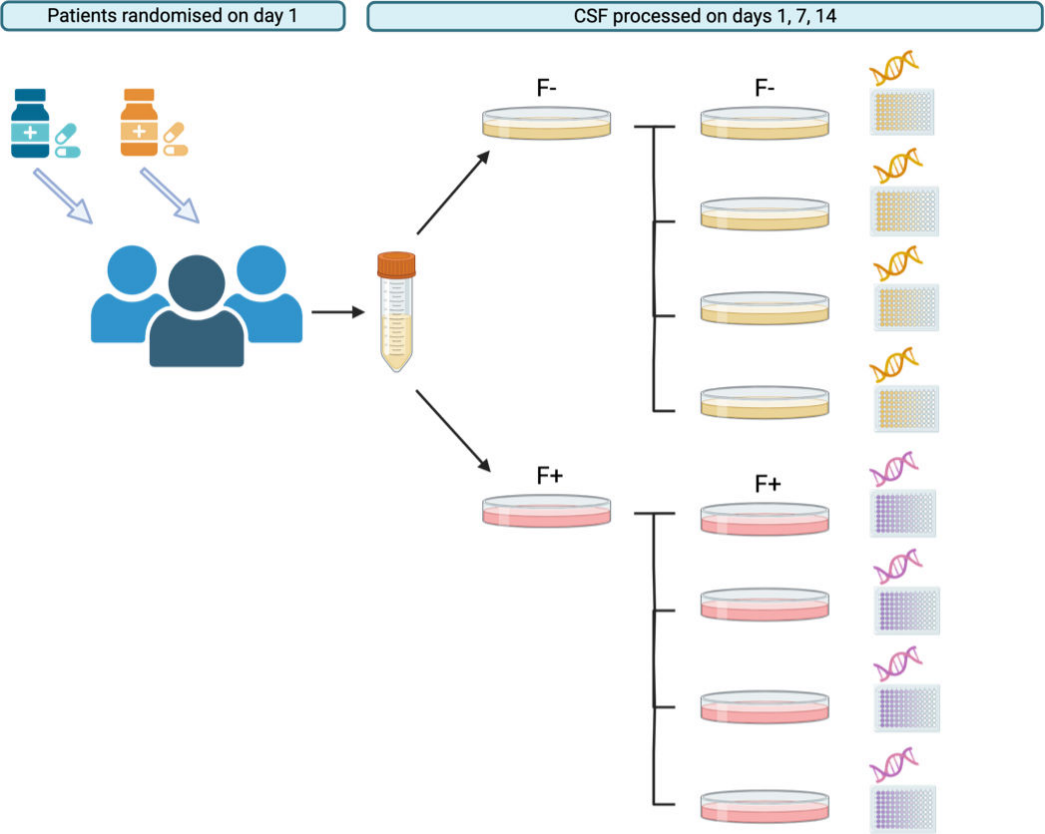
Cerebrospinal fluid processing strategy. Following patient recruitment and randomization, lumbar punctures were performed on days 1 (prior to treatment initiation), 7, and 14. At each time point, CSF was plated onto agar that contained either no fluconazole (F−) or fluconazole at 10 µg/mL (F+). Plates were incubated for 48 h at 30°C. Four colonies were selected from each plate and each colony was plated onto fresh agar with either no fluconazole (F−) or fluconazole at 10 µg/mL (F+), consistent with the initial plating conditions. Plates were again incubated for 48 h at 30°C. Isolates were stored at −80°C until retrieved for WGS and drug susceptibility testing.

For genotypic and phenotypic analyses, multiple colonies from each CSF sample from each patient were stored (Fig. 1). The data set contained 718 isolates from CSF samples; a mean of 11.2 isolates per patient. Isolates from CSF samples taken on study days 1, 7, and 14 were available for 59, 26, and 9 patients, respectively, with a mean of 7 isolates per patient per time point. Where isolates were unavailable for certain time points, this was due to either lack of clinical sample availability (for example, due to patient death) or insufficient or no growth from the sample—particularly on days 7 and 14 after the initiation of antifungal therapy.

**Fig 2:**
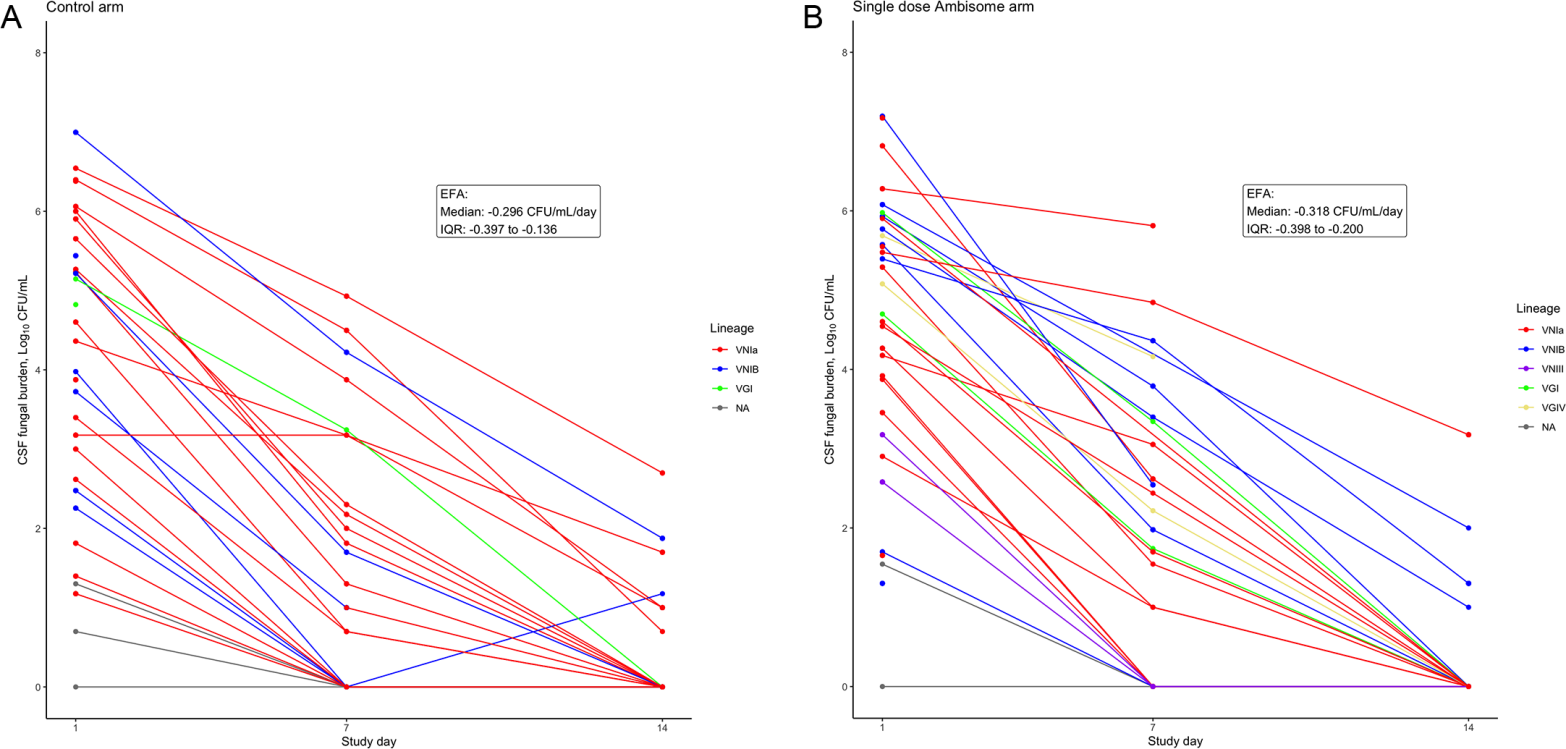
Early fungicidal activity. A decline in fungal burden in 64 patients administered amphotericin-B-based antifungal therapy for HIV-associated cryptococcal meningitis. (A) Control arm; (B) single dose AmBisome arm.

EFA was calculated for all patients where there was >1 quantitative fungal culture available (*n* = 47). Median EFA was −0.298 CFU/mL/day (IQR −0.399 to −0.208; Fig. 2). Mortality was 5/52 (9.6%) at 2 weeks and 13/52 (25.0%) at 10 weeks. In this cohort, the median CSF opening pressure at baseline was 22.0 cm H_2_O (IQR 12.5–32.0 cm).

### *Cryptococcus* isolates sequencing and analysis of strain heterogeneity

Among the 718 isolates, 597 isolates (83%) from 51 patients were identified as *C. neoformans* of the VNI lineage, with 412 (69%) of those being VNIa and 185 (31%) VNIb. In total, 64 isolates (9%) from 4 patients were *C. gattii* (VGI lineage); 40 isolates (5%) from 2 patients were *C. gattii* (VGIV lineage); 12 isolates (2%) from 2 patients were *C. neoformans x deneoformans* hybrids (VNIII lineage) and 5 isolates (<1%) from 2 patients were identified as other yeasts. These were 1 *Candida parapsilosis* isolate from one patient, and 4 *Cryptococcus albidus* isolates from another patient. We removed these other yeasts from further analysis.

**Fig 3:**
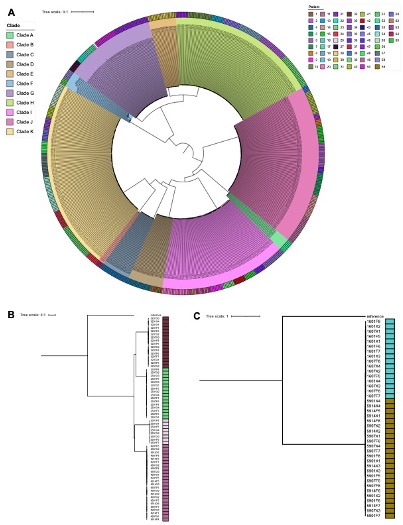
Maximum likelihood phylogeny. Maximum likelihood phylogeny for each lineage found in *Cryptococcus* isolates using RAxML GTRCAT model: (**A**) 585 *C*. *neoformans*, (**B**) 64 *C. gattii* VGI, (**C**) 40 *C. gattii* VGIV. *C. neoformans* isolates annotated by clades or monophyletic groups. All isolates are annotated by the patient of origin.

These were evidence of infection with mixed strains in 3 out of 64 patients: 1 patient contributed 15 VNIb isolates and 1 VNIa isolate, 1 patient contributed 8 VNIa isolates and 4 *C*. *albidus* isolates, and 1 patient contributed 3 VNIb isolates and 1 *C. parapsilosis* isolate. Among the *C. neoformans* isolates, there was evidence of remarkably low strain heterogeneity within individual patients (Fig. 3). Intra-patient isolates harbored a mean of 4 single nucleotide polymorphisms (SNPs) between them at the start of the study (range 1–8). In addition, there was no evidence among these serially collected isolates of any significant evolution of intra-patient strain heterogeneity over time. Isolates were distributed in 11 monophyletic clades, with surprisingly low strain heterogeneity within each clade. Five of the 11 clades were represented by between 6 and 10 patients each, yet exhibited low inter-patient phylogenetic distances. The most genotypically diverse clade was represented by 88 isolates from 9 patients, with mean nucleotide diversity of 1.45E^−05^ and mean inter-patient distance of 276 SNPs (range 66–420 SNPs). The least genotypically diverse clade had a mean nucleotide diversity of 8.74E^−07^ and an interpatient SNP distance of just 25 SNPs (2 patients only). The least diverse clade with greater than 2 patients contained 104 isolates from 7 patients, with mean nucleotide diversity of 4.98E^−06^, and mean inter-patient distance of 93 SNPs (range 16–142 SNPs).

Chromosome 1 aneuploidy was evident in 155 *C*. *neoformans* isolates (26%), and only in those that had been cultured on fluconazole-containing media *ex vivo*. A total of 27 of the 627 isolates (4%) displayed chromosome 12 aneuploidy; 21 displayed chromosome 4 aneuploidy (3%); 14 chromosome 13 aneuploidy (2%), and ≤2 isolates (≤0.3%) displayed aneuploidy of each of chromosomes 2, 6, 7, 8, 9, 10, and 14. Aneuploidy in chromosome 1 and chromosome 12 was stable across all time points, with no evidence of evolution of aneuploidy during infection.

Since we were able to approximate inter-individual variability in drug exposure, we looked for evidence of the evolution of resistance to fluconazole and flucytosine during drug treatment. In total, 319/609 (52%) of isolates had at least one non-synonymous mutation in a drug target gene. Within these isolates, 27 different non-synonymous variants were detected: 11 in AFR1, 7 in ERG11, 2 in FCY1, 4 in FCY2, 2 in FUR2, and 1 in USX1. All of these variants were present in isolates collected on study day 1 and none were lost or gained in samples collected subsequently, suggesting no evolution of resistance during the first 14 days of the trial. Using the phylogeny to infer ancestral states, we determined that there were no recurrent mutations in the non-synonymous drug target gene variants.

### Phenotyping

The impact of chromosome 1 aneuploidy and non-synonymous variants in the above drug target genes of interest on antifungal resistance in *C. neoformans* has been extensively described elsewhere (25, 31–33). We selected the 389 *C*. *neoformans* isolates that were subcultured on fluconazole-free media—i.e., no evidence of chromosome 1 aneuploidy or non-synonymous drug target gene variants—for phenotypic antifungal susceptibility profiling. Initial screen via culture on 32 µg/mL antifungal drug revealed no evidence of phenotypic resistance to fluconazole or flucytosine in any of these isolates. There was also no evidence of phenotypic resistance to fluconazole or flucytosine among the *C. gattii* VGI isolates.

Among *C. gattii* VGIV isolates, there was no evidence of flucytosine resistance. However, all *C. gattii* VGIV isolates were resistant to fluconazole. Among VGIV isolates selected for MIC testing, fluconazole MIC_50_ was 4 µg/mL in 3 isolates, 8 µg/mL in 2 isolates, and 16 µg/mL in 2 isolates.

### Pharmacokinetic models

PK parameter estimates from the final models for each drug are summarized in Table 1. There was considerable inter-individual variability in drug exposure, quantified as the area under the concentration-time curve (AUC) generated in CSF (flucytosine and fluconazole) and plasma (amphotericin B formulations) from each patient’s posterior PK estimates. Summary statistics for the AUC estimates are presented in Table 2. Of note, bioanalysis revealed that fluconazole was detectable in the plasma of 37 of 64 patients (58%) at baseline, before any study-administered fluconazole. The likely explanation for this is that patients were receiving fluconazole for primary prophylaxis of cryptococcal meningitis, or were administered fluconazole upon diagnosis, prior to being enrolled in the Ambition-CM trial. Patients were eligible for the Ambition-CM trial if they had had no more than 2 doses of fluconazole ≥800 mg.

**TABLE 1:** Parameter estimates from the final pharmacokinetic models^*a*^

Parameter	Flucytosine			Fluconazole			Amphotericin B deoxycholate			Liposomal amphotericin B		
	Mean	SD	Median	Mean	SD	Median	Mean	SD	Median	Mean	SD	Median
Ka (h^−1^)	1.77	1.81	1.01	0.63	0.61	0.41	*			*		
CL (L/h)	5.88	3.35	5.31	0.65	0.42	0.57	3.25	1.77	2.48	0.42	0.36	0.35
V (L)	17.5	9.99	14.76	10.44	6.14	10.85	*			4.57	4.52	3.70
K23 (h^−1^)	15.55	4.97	18.57	32.73	32.29	11.67	*			*		
K32 (h^−1^)	9.02	5.43	6.69	9.54	5.37	9.47	*			*		
K24 (h^−1^)	5.68	7.6	1.09	9.92	5.27	9.61	12.50	5.89	11.76	2.22	3.35	0.22
K42 (h^−1^)	1.38	2.33	0.24	7.79	6.20	6.99	8.65	7.92	9.75	2.95	4.07	0.48
Vcns (L)	41.73	13.66	44.55	21.05	8.48	19.98	*			*		
Slope (L/kg)	*			*			0.53	0.40	0.59	*		
Intercept (L)	*			*			3.14	3.93	1.01	*		

^*a*^ Ka, absorption rate constant from gut to central compartment (i.e., circulation); CL, apparent clearance; V, volume of central compartment; K23, first-order rate constant from central compartment to central nervous system (CNS) compartment; K32, first-order rate constant from CNS compartment to central compartment; K24, first-order rate constant from central compartment to peripheral compartment; K42, first-order rate constant from peripheral compartment to central compartment; Vcns, apparent volume of CNS compartment; *, volume was estimated as intercept + slope × patient weight, as previously described (27).

**TABLE 2:** Posterior estimates of AUC for each of the antifungal drugs administered in our patient cohort^*a*^

Drug	Median AUC (mg·L/h)	25th percentile	75th percentile
Fluconazole AUC_144-168_	5,503.62	3,902.53	6,751.9
Fluconazole AUC_0-168_	7,263.37	5,575.57	9,511.55
Flucytosine AUC_144-168_	579.12	343.53	812.94
Flucytosine AUC_0-168_	3,560.05	2,298.65	4,927.89
Amphotericin B deoxycholate AUC_0-24_	18.04	7.03	21.67
Amphotericin B deoxycholate AUC_0-168_	123.01	64.45	173.69
Liposomal amphotericin B AUC_0-24_	540.22	321.2	911.13
Liposomal amphotericin B AUC_0-168_	923.01	408.61	1,383.28
Overall amphotericin B AUC_0-24_	27.64	14.28	540.22
Overall amphotericin B AUC_0-168_	184.84	105.55	923.01

^*a*^ AUC, the area under the concentration-time curve. Fluconazole and flucytosine AUCs were calculated from CSF posterior PK estimates. Amphotericin B deoxycholate and liposomal amphotericin B AUCs were calculated from plasma posterior PK estimates.

### Associations with CSF opening pressure

CSF opening pressure was raised in 56% of the cohort on study day 1 (*n* = 29). After backward selection, baseline fungal burden, presence of at least one non-synonymous mutation in a drug target gene of interest, and sub-lineage VNIb were independently associated with opening pressure. Interestingly, the most significant of these associations was with strain sub-lineage VNIb, which was associated with reduced odds of high opening pressure (greater than 20 cm water; odds ratio of high opening pressure with VNIb strains compared with VNIa strains, 0.29, *P*-value 5.87E^−6^).

### Associations with early fungicidal activity

Several variables were retained in the multivariable regression model that examined predictors of EFA (Table 3). By far the most statistically significant independent association with a 0.1 log change in EFA was amphotericin B AUC_0-24_ (regression coefficient for an increase in amphotericin B AUC_0-24_ of 1 g.L/h = −1.736, *P*-value 5.30E^−17^). EFA was recorded as a negative value, therefore a negative regression coefficient implies an association with increased EFA. Strain lineage was also significantly associated with EFA. Strains of sub-lineage VNIb were associated with increased EFA (regression coefficient for VNIb strains compared with VNIa strains, −0.937, *P*-value 2.56E^−08^), while sub-lineage VNIII strains were associated with decreased EFA (regression coefficient for VNIII strains compared with VNIa strains, 2.236, *P*-value 1.40E^−07^).

**TABLE 3:** Adjusted multivariable regression model examining predictors of early fungicidal activity^*a*^

Association with EFA	Regression coefficient	95% confidence interval	*P*-value
**Arm: single dose**	**0.641**	**0.013, 0.114**	**0.013**
**Lineage VNIb**	**−0.937**	**-1.263, –0.612**	**2.65E** ^**−08**^
**Lineage VNIII**	**2.236**	**1.414, 3.058**	**1.40E** ^**−07**^
Fluconazole AUC_144-168_, g·L/h	0.058	−0.035, 0.152	0.221
**Flucytosine AUC**_**144-168**_ **, g·L/h**	**−0.342**	**-0.524, –0.160**	**2.54E** ^**−04**^
**Amphotericin AUC**_**0-24**_ **, g·L/h**	**−1**.**736**	**-2.130, –1.342**	**5.30E** ^**−17**^
**Baseline log**_**10**_ **CFU**	**−0.162**	**-0.220, –0.104**	**7.00E** ^**−08**^
**Aneuploidy of chromosome 1**	**0.265**	**0.029, 0.501**	**0.028**
Presence of non-synonymous mutation in drug target gene	−0.083	−0.391, 0.224	0.596

^*a*^ Significant associations are highlighted in bold font. CFU, colony-forming units. Note that pharmacokinetic AUC values were modeled as g.L/h rather than mg.L/h for ease of interpretation in the regression model.

### Associations with mortality

In the adjusted multiple regression model, high baseline fungal burden, low EFA, and control/daily study arm (daily dosing of amphotericin B deoxycholate) were significantly associated with mortality at 10 weeks (Table 4). In our cohort, neither strain lineage, aneuploidy of chromosome 1 nor the presence of non-synonymous mutations in drug target genes were associated with mortality. There was no significant association between high versus low lumbar opening pressure and mortality at 10 weeks.

**TABLE 4:** Adjusted multivariable regression model examining predictors of mortality at 10 weeks^*a*^

Variable	Category	Mortality at 10 weeks			
		OR	CI low (2.5%)	CI high (97.5%)	*P*-value
Study arm	Control	1.000			
	Single dose	**0.251**	**0.069**	**0.917**	**0.036**
Baseline fungal burden	1 log increase in CFU	**1.409**	**1.214**	**1.635**	**6.12E^−06^**
Lineage	VNIa	1.000			
	VNIb	>10.000	0.000	NaN	0.984
	VNIII	0.533	0.000	NaN	1.000
Aneuploidy of chromosome 1	Absent	1.000			
	Present	0.601	0.332	1.090	0.093
Amphotericin AUC_0-24_	Increase of 1 g·L/h	1.054	0.379	2.927	0.920
Flucytosine AUC_144-168_	Increase of 1 g·L/h	1.023	0.538	1.948	0.944
Fluconazole AUC_144-168_	Increase of 1 g·L/h	1.803	1.397	2.327	0.058
EFA	More negative by 0.1 log_10_ CFU/mL/day	**0.499**	**0.377**	**0.660**	**1.10E^−06^**
CSF opening pressure	<20 cm H_2_O	1.000			
	>20 cm H_2_O	0.960	0.535	1.723	0.892

^*a*^ Significant associations are highlighted in bold font. OR, odds ratio; CI, confidence interval.

### Associations with aneuploidy of chromosome 1

Since we only detected chromosome 1 aneuploidy in isolates that had been subcultured on fluconazole-containing media, we adjusted for plating conditions when exploring the association of other variables with chromosome 1 aneuploidy. There was no evidence of *de novo* evolution of chromosomal aneuploidy, therefore examination of predictor variables associated with aneuploidy of chromosome 1 was restricted to baseline variables (fungal burden and strain lineage). Aside from the plating condition, lineage VNIb was significantly associated with chromosome 1 aneuploidy (OR 1.69 in comparison to VNIa, *P*-value 0.04). In the adjusted model, baseline CSF fungal burden was not significantly associated with chromosome 1 aneuploidy.

### Prediction of non-synonymous variants

Similarly, there was no evidence of *de novo* evolution of non-synonymous variants in genes that are associated with resistance to fluconazole or flucytosine so we only examined baseline variables for their association with non-synonymous variants. In the adjusted model, only fungal burden was associated with the detection of at least one non-synonymous variant (regression coefficient 0.35, *P*-value 4.35E^−7^).

## DISCUSSION

We have presented an analysis that is unique in scope, integrating pathogen, pharmacokinetic, and clinical data collected across serial time points in a cohort of patients undergoing treatment for HIV-associated cryptococcal meningitis. We have provided novel insight into the relative contribution of pathogen genomic characteristics, including those associated with antifungal drug resistance, and individual-level estimates of drug exposure, on population-level heterogeneity in clinical outcomes from this disease.

The majority of isolates in our study were of the VNI lineage, which is globally distributed with a largely clonal population structure (34–36). This is in keeping with other studies from Malawi, which have reported a predominance of VNI strains (37, 38). Within the VNI lineage, we identified a majority belonging to VNIa (69% of VNI and 57% of all isolates), compared with 98.5% reported by Ashton et al among isolates collected from five countries in Asia and Africa (38), and 30% reported by Desjardins et al. among isolates collected primarily from Botswana (6). Since the majority of studies of cryptococcus genomics are performed on single-colony isolates, the possibility of infection with mixed strains has mainly been speculative to date (37, 39). However, where investigators have examined multiple colonies from a single patient sample, mixed infections have been reported. Among 13 patients in the Ivory Coast, sero-genotyping and multi-locus sequence typing (MLST) of 252 cryptococcal isolates revealed that 4/13 patients (31%) had infections with mixed sequence types (40). Moreover, serial sampling revealed the evolution of strain diversity at the species and/or sequence type level over time (40). Another study that analyzed 100 isolates from 49 patients using MLST revealed mixed infections—defined as mixed mating types, serotypes, and/or genotypes—in 18.5% of patients (41). A striking observation from our bioinformatic analysis is the genotypic homogeneity among our clinical isolates within individual patients, over three distinct time points. There were just three occurrences of mixed infection (<5% of patients). The mean pairwise difference between genomes in our study was 2 orders of magnitude lower than that reported in other VNI isolates from Malawi (42), and the mean SNP distance we report here is at the lower end of the range previously reported in VNI strains (38). We collected 8 colonies at up to 3-time points in 59 patients and would have expected to detect strain diversity if it were present. Environmental sampling and detailed data concerning migration and population movement in and around Blantyre may elucidate this finding further.

Heteroresistance is an intrinsic feature of *C. neoformans*, with aneuploidy of chromosome 1 as the predominant mechanism (21, 22, 43). Aneuploidy was present in over a quarter of our isolates at baseline, in keeping with other clinical data (21). There was no evidence of evolution of chromosome 1 aneuploidy during the study. More than half of the isolates harbored non-synonymous variations in drug target genes, but again there was no evidence that such variations evolved during the 14-day sampling period. Prior clinical work has demonstrated that it is possible to detect increases in heteroresistant colonies during 14 days of antifungal treatment with fluconazole monotherapy (21). The reason that we did not detect any such increase may be that all of these patients were administered combination antifungal therapy with highly potent, amphotericin B-based antifungal drug regimens. The rapid reduction in fungal burden may have reduced the opportunity for the evolution of genomic features that are associated with antifungal resistance. In addition, the combination of flucytosine with fluconazole suppresses the expansion of heteroresistance in *Cryptococcus* spp. (21), which may have provided further protection for patients in the investigational study arm.

VNI strains have been associated with the production of microcells, which can augment central nervous system (CNS) dissemination and present with features of increased intracranial pressure (15). However, this has not been assessed at the sub-lineage level to our knowledge. In our study, aneuploidy of chromosome 1, reduced odds of high lumbar opening pressure, and EFA were more closely associated with the VNIb sub-lineage than the VNIa sub-lineage. Within VNIa, the VNIa-93 sub-clade is predominant in Malawi and is associated with a reduced risk of death compared with other sub-clades (VNIa-4 and VNIa-5) (38). We are not aware of data comparing VNIa to VNIb in terms of clinical outcomes and further research in this area may highlight avenues for potential therapeutic targets.

Our data is consistent with others that have associated increased EFA with improved mortality outcomes from HIV-associated cryptococcal meningitis (3, 5). In the present analysis, baseline fungal burden, study arm, and EFA were the only variables that retained a significant association with mortality in the adjusted analysis. Neither strain lineage nor genotypic features associated with antifungal resistance retained statistical significance in the adjusted model, though the relative lack of strain diversity may have limited our power to detect associations with mortality. To mitigate the development of a high baseline fungal burden, early diagnosis, and effective antifungal therapy are key. At the point of symptomatic cryptococcal meningitis, our data underscore the importance of optimized EFA. The variable that was most significantly associated with EFA in our analysis was exposure to amphotericin B. We have previously presented population PK models that demonstrate considerable variability in exposure to amphotericin B deoxycholate (27) and liposomal amphotericin B (30) among patients with HIV-associated cryptococcal meningitis given the same weight-based dose of either formulation. In the present analysis, we have taken a novel approach that suggests the relative importance of drug exposure in driving EFA in these patients, compared with genotypic attributes.

The reasons that neither flucytosine nor fluconazole AUC feature in the adjusted model examining predictors of EFA may be twofold. First, amphotericin B formulations are more potent than flucytosine or fluconazole on a per-mg basis and the PD effect of amphotericin B may therefore mask that of the oral drugs in the adjusted model. Second, the PD effect of flucytosine is thought to be driven by the percentage of the dosing window that drug levels are greater than the MIC of the infecting organism (%T > MIC). We did not have flucytosine %T > MIC for these patients so AUC was used as a relevant approximation of drug exposure.

In our data, amphotericin B AUC was associated with EFA, and EFA was associated with a reduction in mortality, but amphotericin B AUC was not directly associated with a reduction in mortality. While these statements appear contradictory, there are multiple reasons why they likely all represent reality. As amphotericin B exposure increases, fungicidal activity in the CNS increases and so does EFA. However, as amphotericin B exposure increases so too does the risk of drug toxicity (27). Amphotericin B-induced nephrotoxicity is multifaceted and dose-dependent, involving direct tubular toxicity and alterations in renal blood flow (44, 45). Electrolyte derangements increase the risk of cardiotoxicity (45–47). Anemia occurs due to suppression of erythropoiesis (45). Both the nephrotoxicity and the anemia induced by amphotericin B increase the odds of mortality at 10 weeks (44). The possibility that amphotericin B toxicity may be a key contributor to mortality in our patient cohort is supported by the fact that the odds of mortality were lower in the single dose arm (single dose of liposomal amphotericin B) than they were in the control arm (7 daily doses of the more toxic amphotericin B deoxycholate), despite lack of direct association between amphotericin B AUC and mortality.

In addition, there are factors aside from EFA that may not be represented in our data, but which contribute to mortality in patients with HIV-associated cryptococcal meningitis. These may be related to the patient and/or disease process; older age, altered mental status, anemia, and low body weight are all independent predictors of mortality (3). The complex immune dysregulation that is a feature of HIV-associated cryptococcal meningitis may increase mortality risk independent of EFA (48–50). Alternatively, there may be factors unrelated to cryptococcal meningitis itself; for example, these profoundly immunosuppressed patients may suffer from concomitant bacteraemia, malnutrition, disseminated mycobacterial and viral infections, and non-cryptococcal invasive fungal diseases (51–53).

This study is limited by the relatively small number of participants relative to the number of isolates analyzed. In addition, we necessarily relied on posterior estimates of drug exposure, based on pharmacokinetic models with their inherent limitations that we have discussed elsewhere (27–30). Nevertheless, our study successfully integrates genomic and pharmacokinetic variables with clinical outcome data in a cohort of patients with advanced HIV disease and cryptococcal meningitis. We report on a surprising level of genomic homogeneity in cryptococcal isolates from this cohort of Malawian patients. Despite this, there was a heterogenous EFA response between patients and this was associated with mortality. *C. neoformans* sub-lineage VNIb was independently associated with a number of clinical variables. The most significant association with EFA in our data was the level of amphotericin B exposure. Future studies may consider the evaluation of dosing strategies that further increase amphotericin B exposure, for example through higher doses of liposomal amphotericin B. In the current era of novel anti-cryptococcal agents emerging from the development pipeline, this study underscores the importance of optimal exposure to potent antifungal drugs to promote improved clinical outcomes.

## MATERIALS AND METHODS

### Clinical study

The current study was conducted as a substudy of the phase III Ambition-CM trial (2). Patients in Ambition-CM were randomized in a 1:1 ratio to receive induction antifungal therapy with either a single 10 mg/kg dose of liposomal amphotericin B (AmBisome, Gilead Sciences) alongside 14 days of flucytosine (100 mg/kg/day) plus fluconazole (1,200 mg/day), or amphotericin B deoxycholate (1 mg/kg/day) plus flucytosine (100 mg/kg/day) for 7 days, followed by fluconazole (1,200 mg/day) on days through 14. All participants eligible for Ambition-CM were eligible for the current substudy; substudy recruitment proceeded opportunistically with serial Ambition-CM participants.

Lumbar opening pressure was recorded on day 1. Quantitative CSF cryptococcal cultures were performed on days 1, 7, and 14 (2).

### Pharmacokinetic samples

Drug administration times were documented in real-time by patients or caregivers. For PK samples, a volume of 2 mL of blood was collected into heparinized collection tubes and placed on ice. CSF was collected into sterile containers. Within 30 min of collection, samples were centrifuged at 1,500 *× g* for 10 min at 4°C. Plasma and CSF supernatant were stored at −80°C until bioanalysis.

### Culture and storage of cryptococcal isolates

CSF was plated within 1 h of collection onto Sabouraud dextrose agar (SAB). Every sample from every patient was plated in duplicate: onto SAB with no antifungal drugs added and onto SAB containing fluconazole at a concentration of 10 µg/mL. Plates were incubated for 48–72 h at 30°C. Four colonies were selected at random from each of these plates and subcultured on SAB with conditions consistent with the initial culture. These subcultures were again incubated for 48 h at 30°C and the resulting colonies were stored on beads at −80°C. The isolate processing strategy is represented in Fig. 1.

### DNA isolation and whole genome sequencing

Isolates were grown in 3.5 mL of 2× YPD broth, with or without fluconazole 10 µg/mL. Cells were harvested, washed once with 1 mL ddsH_2_O, and pelleted before 8–12 2.3 mm diameter silica beads were added. Samples were frozen at −80°C and lyophilized overnight. Genomic DNA was isolated using the Masterpure Yeast DNA purification kit (LGC Biosearch Technologies). Briefly, 300 µL of the yeast cell lysis solution was added to the lyophilized samples, followed by bead milling twice at 5,500 rpm for 30 s intervals at 4°C. Next, 5 mL of 20 µg/mL RNase A (New England Biolabs) was added to each sample vortexed briefly, and incubated for approximately 2 h at 65°C. Samples were cooled on ice and 150 µL of MCP protein precipitation reagent was added, vortexed for less than 10 s, and centrifuged at 11,000 *× g* for 15 min. The supernatant was added to 500 µL of ice-cold isopropanol and inverted to mix. Samples were centrifuged for 10 min at 11,000 × *g*. The precipitated DNA was washed twice with 0.5 mL of 70% ethanol. The pellet was air-dried for 2–5 min and the DNA was resuspended in sterile double-distilled water.

Library preparation was performed on 3.3–16.7 ng/µL of extracted genomic DNA from all isolates using the Illumina DNA prep (Nextera Flex) library prep kit according to the manufacturer’s instructions. Samples were sequenced on an Illumina NovaSeq 6000 platform using an S2 full-flow cell to obtain paired-end 100 bp reads.

### Read alignment and variant identification

Identification of the *Cryptococcus* species for each sample was determined by alignment to a pan-genome containing representatives of the *C. gattii* species complex (VGI, VGII, VGIIIa, VGIIIb, VGIV), *C. neoformans* (H99), and *C. deneoformans* (JEC21) using Burrows-Wheeler Aligner (BWA) MEM v0.7.17 and determining the highest percentages of reads mapping to a single reference genome (GenBank assembly accession [https://www.ncbi.nlm.nih.gov/datasets/genome/], *Cryptococcus neoformans* var. grubii H99 GCF_000149245.1, *Cryptococcus neoformans* var. neoformans (*deneoformans*) JEC21 GCF_000091045.1, VGI *Cryptococcus gattii* WM276 GCF_000185945.1, VGII *Cryptococcus gattii* (*deuterogattii*) VGII R265 GCA_002954075.1, VGIIIa *Cryptococcus gattii* (*bacillisporus*) CA1873 GCA_000855695.1, VGIIIb *Cryptococcus gattii* (*bacillisporus*) CA1280 GCA_000836335.1, VGIV *Cryptococcus gattii* (*tetragattii*) IND107 GCA_000835755.1, VGV *Cryptococcus cf. gattii* MF34 GCA_009650685.1). Paired-end reads from isolates identified as *Cryptococcus neoformans* (H99) or *Cryptococcus gattii* (VGI, VGIV) were aligned to only the respective reference genomes using BWA MEM v0.7.17.

Isolates that did not align well with the *Cryptococcus* pangenome were *de novo* assembled using SPAdes v3.11.1. The draft assemblies were initially compared to the Targeted host-associated Fungi ITS database (THFv1.6.1) to map species using BLASTN v2.9.0+. The contigs from the draft assembly were then aligned to the respective reference genomes to confirm high identity genome matching using the nucmer program from the MUMmer3.23 package (GenBank assembly accession: *Cryptococcus albidus* GCA_001599735.1, *Candida parapsilosis* GCA_036288975.1). The average nucleotide identity calculated using the GAEMR (http://software.broadinstitute.org/software/gaemr/) package was >97% for *C. albidus* and >99% for *C. parapsilosis*.

Variants were called in accordance with the pipeline detailed in the following github repository: https://github.com/broadinstitute/fungal-wdl/tree/master/gatk4. Variants were then identified using GATK v4.1.8.1 using the haploid mode and preprocessed using GATK tools (54). Variants were filtered with VariantFiltration if QualbyDepth < 20.0, FisherStrand > 60.0, or RMSMappingQuality < 40.0. Genotypes were filtered if the minimum Genotype Quality < 50.0, the minimum percent alternate allele in Allelic Depth < 0.8, or the minimum total Depth < 10.0, using a custom Python script (https://github.com/broadinstitute/broad-fungalgroup/tree/master/scripts/SNPs).

Maximum likelihood phylogenetic trees were estimated for the isolates identified as H99, VGI, and VGIV using segregating SNP sites present in one or more isolates, allowing ambiguity from SNP variant sites in a maximum of 10% of samples, using RAxML v7.7.8 with model GTRCAT (55). Aneuploidies were first evaluated using funpipe (coverage analysis) v0.1.0 (https://github.com/broadinstitute/funpipe) and then copy number variation regions were called using CNVnator v0.3 (56). The following known drug target genes were assessed for non-synonymous variants: ERG11, AFR1, FUR1, FCY1, FCY2, and UXS1. All variants were annotated with SNPeff, v4.1g. Recurrent drug target mutations that appeared after the diversification of the 11 clonal clades were evaluated by ancestral state reconstruction using TreeTime v0.11.1 with “–method_anc probabilistic” with the reference tree of 583 CnAH99 isolates and predicted variants in drug target genes. Variants were considered “recurrent” if they were assigned to more than one internal node.

### Phenotyping

Strains were prepared from freezer stocks and grown on YPD for 72 h at 30°C. A single colony from each isolate was inoculated into a 96-well plate containing 250 µL of YPD broth and incubated for 24–48 h days at 30°C. Each 96-well plate contained control strains (H99 and fluconazole-resistant strain [DUMC118.00]), a 5FC resistant mutant strain (*CNAG_05076*∆), and its parental background strain (CM026). Next, cells were pin replicated from the 96-well plates into 384-well microtiter plates containing 80 µL of YPD broth, incubated for 24–48 h at 30°C and pinned in a 1,536 array format using the BM5-BC Colony Processing Robot (S & P Robotics) onto media in a one-well rectangular plate (Nunc OmniTray, Thermo Fisher Scientific). Strains were arrayed onto the following media conditions: YPD containing 0 or 32 µg/mL fluconazole (Thermo Fisher Scientific) and YNB containing 0 or 32 µg/mL flucytosine (Thermo Fisher Scientific). Thus, each strain was represented by 16 colonies in a 4 × 4 arrangement for each 1,536 array. Array plates were incubated at 30°C and images were acquired daily for 5 days. Three independent experiments were performed.

We identified isolates with drug-resistant growth phenotypes via automated image analysis with CellProfiler (57). A modified version of the CellProfiler YeastPatches pipeline was used to measure the growth area for each colony in the 1,536-spot array plates. To account for errors in colony detection, the two largest and two smallest colony area measurements for each isolate were discarded before calculating the average colony area per isolate. A minimum threshold of 4,000 px average colony area was chosen to classify growth as drug-resistant, based on average area measurements of drug-resistant control isolates. Isolates with greater than 4,000 px average colony area in all replicate plates were reported as drug-resistant.

### Antifungal susceptibility testing

Broth microdilution susceptibility testing was performed in duplicate on selected *Cryptococcus* isolates and strains according to the Clinical and Laboratory Standards Institute guidelines for M27-A3 (58). Fluconazole was diluted in microtiter plates to obtain a final concentration of 64 to 0.5 µg/mL. Plates were incubated for 72 h at 35°C. The minimum concentration that resulted in a 50% reduction in yeast growth compared to the untreated control was determined and defined as the MIC.

### Pharmacokinetic bioanalysis

Bioanalytical procedures for measurement of liposomal amphotericin B and flucytosine are described elsewhere (29, 30). For bioanalytical methods for fluconazole and amphotericin B deoxycholate, see Supplemental material.

### Pharmacokinetic modeling

We modeled the concentration-time data for fluconazole, flucytosine, and amphotericin B in plasma and CSF using the nonparametric adaptive grid algorithm of the program Pmetrics (59) version 2.0.2, for R version 4.2.0. For flucytosine and liposomal amphotericin B, we used the population PK models that we previously constructed using these same data (29, 30). For fluconazole and amphotericin B deoxycholate, new models were fit according to the same structure as we have previously published using different data sets (27, 28).

Using the final population PK models, individual patients’ exposure to each drug was estimated by calculating the AUC during the first 24 h (AUC_0-24_; for the amphotericin formulations) or at steady state (AUC_144-168_; for fluconazole and flucytosine), as well as overall exposure during the first week of treatment (AUC_0-168_; all drugs) using the Bayesian posterior PK predictions for each patient to perform trapezoidal approximation in Pmetrics.

### Statistics

Data were analyzed using R version 4.2.0. EFA was calculated by fitting a linear regression of log_10_ CFU per mL CSF during the first 14 days of antifungal therapy. Univariable analyses were conducted to investigate potential relationships between independent variables and categorical dependent variables using logistic regression, chi-squared, or Fisher exact tests. Potential relationships between independent variables and continuous dependent variables were investigated using linear regression, analysis of variance, or *t*-tests. Statistical significance was defined as *P*-value < 0.05. Baseline fungal burden was included in all multivariable models a-priori, and baseline fungal burden and EFA were included in models exploring associations with mortality a-priori.

## Data Availability

Isolate sequence data can be accessed in NCBI via accession number PRJNA1103328. The library_ID included in this data includes a sequence of 6 digits, which represent the patient number, the day of sample collection, and the condition in which the isolate was cultured. For example, "0107F2" represents an isolate collected from patient 1 on day 7 that was cultured on fluconazole-containing media. "2401X3" is an isolate from patient 24 collected on day 1 and cultured on fluconazole-free media.

## SUPPLEMENTAL MATERIAL

Supplemental material mbio.01592-24-s0001.docx Additional details of authorship and methods.

ASM does not own the copyrights to Supplemental Material that may be linked to, or accessed through, an article. The authors have granted ASM a non-exclusive, world-wide license to publish the Supplemental Material files. Please contact the corresponding author directly for reuse.
