# Supplementary material for: Integration of genomic and pharmacokinetic data to predict clinical outcomes in HIV-associated cryptococcal meningitis
Source: mBio. 2024 Aug 27;15(10):e01592-24. doi: 10.1128/mbio.01592-24 (PMC11481554; doi:10.1128/mbio.01592-24)
Supplement: Supplemental material — Additional details of authorship and methods. [file mbio.01592-24-s0001.docx]

**Supplemental Material**

The AMBITION Study Group

In addition to the named authors, the following were members of the Ambition Study Group:

Malawi-Liverpool-Wellcome Trust Clinical Research Programme / Queen Elizabeth Central Hospital, Blantyre, Malawi –T Chimphambano, E Dziwani, A Kadzilimbile, S Kateta, E Kossam, C Kukacha, B Lipenga, J Ndaferankhande, M Ndalama, R Shah, A Singini and A Zambasa.

Botswana Harvard AIDS Institute Partnership / Princess Marina Hospital, Gaborone, Botswana – J Goodall, K Lechiile, N Mawoko, T Mbangiwa, J Milburn, R Mmipi, C Muthoga, P Ponatshego, I Rulaganyang, K Seatla, N Tlhako and K Tsholo.

University of Cape Town / Mitchells Plain Hospital / Khayelitsha District Hospital, Cape Town, South Africa – S April, A Bekiswa, L Boloko, H Bookholane, T Crede, L Davids, R Goliath, S Hlungulu, R Hoffman, H Kyepa, N Masina, D Maughan, T Mnguni, S Moosa, T Morar, M Mpalali, J Naude, I Oliphant, S Sayed, L Sebesho, M Shey and L Swanepoel.

UNC Project, Kamuzu Central Hospital, Lilongwe, Malawi – T Banda, T Chikaonda, G Chitulo, L Chiwoko, N Chome, M Gwin, T Kachitosi, B Kamanga, M Kazembe, E Kumwenda, M Kumwenda, C Maya, W Mhango, C Mphande, L Msumba, T Munthali, D Ngoma, S Nicholas, L Simwinga, A Stambuli, G Tegha and J Zambezi.

Infectious Diseases Institute / Kiruddu General Hospital, Kampala, Uganda – C Ahimbisibwe, A Akampurira, A Alice, F Cresswell, J Gakuru, D Kiiza, J Kisembo, R Kwizera, F Kugonza, E Laker, T Luggya, A Lule, A Musubire, R Muyise, O Namujju, J Ndyetukira, L Nsangi, M Okirwoth, A Sadiq, K Tadeo, A Tukundane and D Williams.

Infectious Diseases Institute / Mbarara Regional Referral Hospital, Mbarara, Uganda – L Atwine, P Buzaare, M Collins, N Emily, C Inyakuwa, S Kariisa, J Mwesigye, S Niwamanya, A Rodgers, J Rukundo, I Rwomushana, M Ssemusu and G Stead.

University of Zimbabwe / Parirenyatwa General Hospital, Harare, Zimbabwe – K Boyd, S Gondo, P Kufa, E Makaha, C Moyo, T Mtisi, S Mudzingwa, T Mwarumba and T Zinyandu.

Institut Pasteur, Paris, France – A Alanio, F Dromer and A Sturny-Leclere.

London School of Hygiene and Tropical Medicine, London, UK – P Griffin and S Hafeez.

Pharmacokinetic bioanalysis – fluconazole and amphotericin B deoxycholate

Fluconazole was extracted from human plasma and analysed as follows. The internal standard, [2H4] fluconazole (Alsachim, France) was prepared in acetonitrile (1 mg/L, Fisher Scientific UK) and 100 µL was added to a 96-well protein precipitation plate [Phenomenex, Cheshire, UK]. Twenty-five µL each of human plasma samples, blanks, calibrators in the range 0.1 – 100 mg/L and quality controls (0.75, 7.5 and 75 mg/L) was mixed with the internal standard on an orbital shaker for 5 mins. For amphotericin B deoxycholate, the internal standard natamycin (Cambridge Biosciences, UK) was prepared in methanol (1 mg/L, Fisher Scientific UK) and 200 µL was added to a 96-well protein precipitation plate [Phenomenex, Cheshire, UK]. Fifty µL each of human plasma samples, blanks, calibrators in the range 0.025 – 10 mg/L and quality controls (0.05, 0.75 and 7.5 mg/L) was mixed with the internal standard on an orbital shaker for 5 mins. For both compounds, liquid was drawn through the protein precipitation plate into a collection plate using a positive pressure manifold. Water and 0.1% formic acid (1000 µL) were added to each well. The plate was sealed and placed onto an orbital shaker for 5 mins before being transferred to the autosampler for analysis by LC-MS-MS.

LC-MS-MS analysis was carried out using a Waters Acuity UPLC coupled to a Waters Xevo TQ-XS triple quadrupole mass spectrometer fitted with an electrospray source. The LC-MS system was controlled using MassLynx Security Data Acquisition software (Ver 4.2). Analytes were injected (2 µL) onto a Waters HSS T3 100 Å column (2.1 mm x 100 mm, 1.8 µm, 40°C) and separated over a 3.5 min. gradient using a mixture of solvents A and B. Solvent A was LC-MS grade water with 0.1% (v/v) formic acid. Solvent B was HPLC grade acetonitrile with 0.1% (v/v) formic acid. Separations were performed by applying a linear gradient of 5% to 95% solvent B over 2.5 mins at 0.4 mL/min followed by an equilibration step (1.0 mins at 5% solvent B).

The mass spectrometer was operated in positive ion mode using a Multiple Reaction Monitoring (MRM) method. Following an optimisation process the following mass transitions and collision energies were used for the analysis. Fluconazole: 307.0 > 220.11 (Ce 16 ev) and 311.2 > 223.0 (Ce 16 ev). Amphotericin B: 924.6 > 743.5 (Ce 20 ev) and 666.5 > 503.3 (Ce 16 ev). Data were processed using TargetLynx XS within the MassLynx Security software (Ver 4.2). Assay validation and sample analysis were carried out in accordance with FDA Bioanalytical method validation guidelines for clinical samples.(61)
